# Managing Cow’s Milk Protein Allergy in Indonesia: A Cost-effectiveness Analysis of Hypoallergenic Milk Formulas from the Private Payers’ Perspective

**DOI:** 10.36469/001c.36407

**Published:** 2022-09-07

**Authors:** Ana Teresa Paquete, Rui Martins, Mark P. Connolly, Badriul Hegar, Zakiudin Munasir, Stephanus Stephanus

**Affiliations:** 1 Global Market Access Solutions, St-Prex, Switzerland; 2 Department of Pediatrics, Faculty of Medicine University of Indonesia; 3 Division of Allergy and Clinical Immunology, Faculty of Medicine University of Indonesia; 4 Mead Johnson Indonesia

**Keywords:** cow’s milk protein allergy, hypoallergenic formula milk, atopic march, cost-effectiveness, Southeast Asia

## Abstract

**Background:** Cow’s milk protein allergy is very common in early childhood. Extensively hydrolyzed formulas are recommended in the first-line management of cow’s milk protein allergy in non-breastfed children. Choice of formulas should be informed by efficacy and cost data. **Objectives:** This study aims to compare the cost-effectiveness of extensively hydrolyzed casein formula with *Lacticaseibacillus rhamnosus* Gorbach Goldin (EHCF+LGG), extensively hydrolyzed whey formula (EHWF), amino acid formula, and soy formula in the first-line management of cow’s milk protein allergy in non-breastfed children in Indonesia. **Methods:** A trial-based decision analytic cohort model was adapted to simulate the occurrence of cow’s milk protein allergy symptoms or being symptom free. The model was based on a prospective nonrandomized study that followed up children for 36 months. Costs and health consequences were discounted at 3% annually. Resources required to manage cow’s milk protein allergy and unit costs for clinical appointments and exams were based on a panel of 15 clinicians, from a private payers’ perspective. Other unit costs were based on publicly available national data. Results were reported as cost per additional child free from allergic manifestations or per additional immunotolerant child at 3 years, and per life-years under the same conditions. Uncertainty was assessed using deterministic and probabilistic sensitivity analysis. **Results:** Children receiving EHCF+LGG were associated with more symptom-free time, a higher probability of cow’s milk tolerance at 3 years, and lower healthcare resources and transportation use when compared with children receiving other formulas (with 38%-49% lower costs). Formula costs were lower for soy, but EHCF+LGG was predicted to save 9% and 54% of overall costs compared with extensively hydrolyzed whey formula and amino acid formula, respectively. Results were robust to sensitivity analyses. **Conclusion:** Use of EHCF+LGG resulted in more symptom-free time and the highest 3-year probability of cow’s milk tolerance. It also led to healthcare resource and transportation savings when compared with other hypoallergenic milk formulas. Soy formula remained an alternative if formula price represents a major constraint.

## BACKGROUND

Cow’s milk protein allergy (CMPA) is one of the most common food allergies worldwide, typically presenting in the first year of life.^1,2^ The prevalence of CMPA in Indonesia remains unknown; however, literature reviews have reported no difference in CMPA prevalence rates between Asian and Western populations.^3,4^ It is thought that CMPA affects 0.5% to 3.0% of infants, although there is substantial variability introduced by predefined diagnostic criteria and common misdiagnosis.^3,5,6^ In studies using self-reported criteria, prevalence ranges from 1.2% to 17.0%.^3,7^ Allergy to cow’s milk protein (CMP) manifests in a range of gastrointestinal, dermatological, and respiratory symptoms that can be detrimental to children’s nutritional status and development, leading to unnecessary health costs.^5,8^ Food allergies occurring early in life can also contribute to the atopic march, increasing the risk of asthma and allergic rhinitis later in life.^9,10^ CMPA is often categorized into immunoglobulin E (IgE)–mediated and non-IgE-mediated symptoms. IgE-mediated reactions consist of allergic manifestations (AM) occurring within 1 to 2 hours of allergen ingestion. Non-IgE symptoms present within hours to days.^10,11^ Indonesian and other international guidelines recommend the use of an extensively hydrolyzed formula in the first-line management of CMPA in non-breastfed children.^3,8,12,13^ Because several casein and whey formulations are available, product choice should be informed by efficacy and cost data, ensuring the best use of resources.

One prospective study found extensively hydrolyzed casein formula (EHCF) with or without *Lactobacillus rhamnosus* Gorbach Goldin (LGG) (now renamed *Lacticaseibacillus rhamnosus*)^14^ added to the matrix associated with higher probability of cow’s milk tolerance compared with rice hydrolyzed formula (RHF), soy formula (SF), and amino acid–based formula (AAF) at the 12-month follow-up.^15^ At a later stage, a randomized control trial recruiting children with IgE-mediated CMPA found that EHCF+LGG was associated with a 23% reduction in AM and a 20% higher probability of becoming cow’s milk–tolerant at 36 months compared with EHCF alone.^16^ A recent publication of a prospective cohort study reports that, after 3 years, children receiving EHCF+LGG were statistically significantly less likely to have any AM and had a higher probability of being tolerant to cow’s milk than children receiving different formulas.^17^

Previous health economic analyses have explored the cost-effectiveness of hypoallergenic formulas in managing infants with CMPA in Italy, Spain, Poland, and the United Kingdom (UK), but none have used head-to-head comparative data over a 36-month period in Indonesia.^18–22^ The objective of this economic evaluation was to assess the cost-effectiveness of commonly used hypoallergenic milk formulas in infants and young children presenting with IgE-mediated CMPA in Indonesia (AAF, EHCF+LGG, extensively hydrolyzed whey formula [EHWF], and SF), applying the most recent evidence in the field. We have considered information on healthcare resource utilization in several cities in Indonesia. The study considers the perspective of private payers only. The Consolidated Health Economic Evaluation Reporting Standards (CHEERS) approach for reporting economic evaluations was used to prepare this work.^23^

## METHODS

### Model Structure

A previously published trial-based decision analytic cohort model was adapted to simulate the use of hypoallergenic formulas to manage IgE-mediated CMPA in non-breastfed children in Indonesia.^22^ The model structure was primarily published on a cost-effectiveness analysis in the UK.^22^ Data from a prospective trial directly comparing AAF, EHCF+LGG, EHWF, and RHF were used to inform annual probabilities of AM and of acquiring immunotolerance to CMP over the 3-year time horizon of the analysis.^17^ To follow children during the period in which they most often develop CMPA symptoms, the study considered a time horizon of 3 years. Although it is our understanding that achieving immune tolerance will benefit adult healthcare, this was not modeled in the current study.

We have not included RHF in the analysis as these are not available in the Indonesian market nor recommended by the Indonesian Pediatric Association for first-line management of CMPA.^12,13^ Soy formula was included in the analysis because it is recommended if extensive hydrolyzed formulas are not available or if formula price constrains access to hypoallergenic milk. The Indonesian Pediatric Society highlights that the incidence of soy protein allergy in infants ranges from 10% to 20%, reaching 25% in infants under 6 months of age and 5% of infants over 6 months.^12^

**Figure 1.**
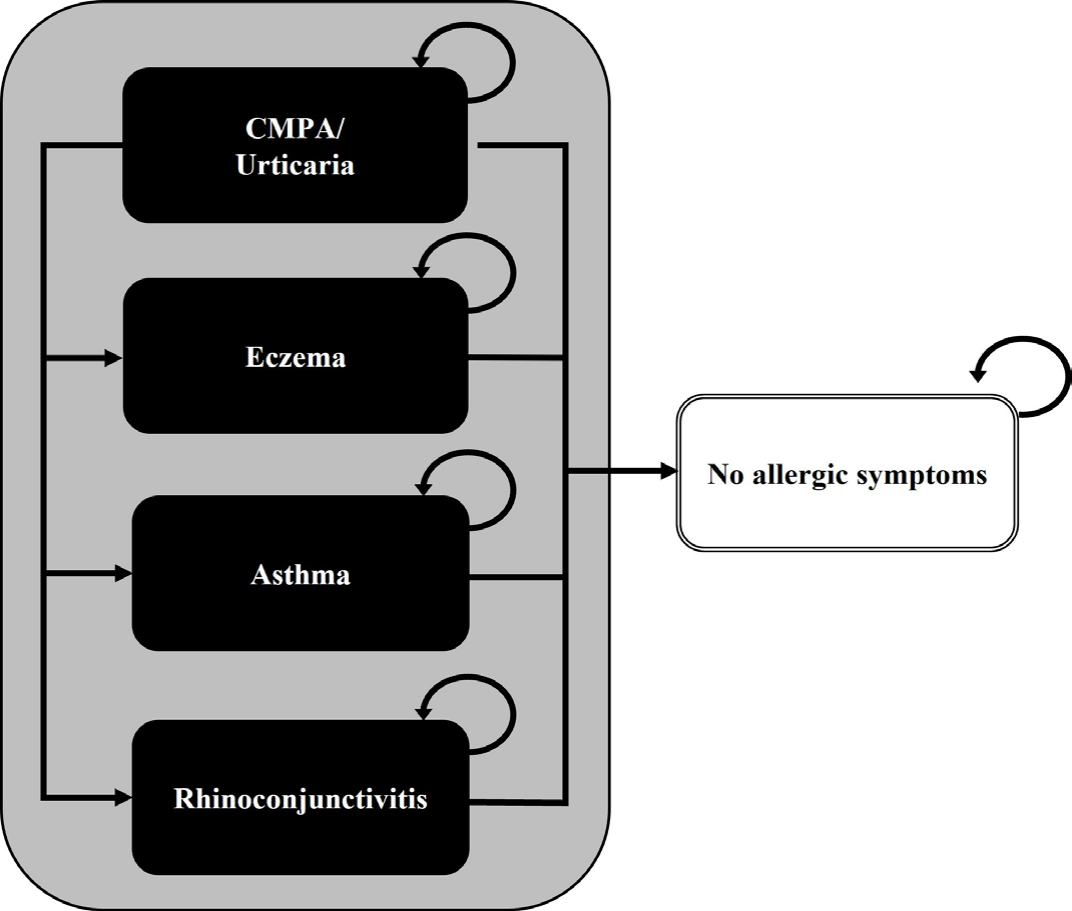
Model Structure Source: Martins R, Connolly M, Minshall E. *J Health Econ Outcomes Res*. 2021;8(2):14-25.^22^ Abbreviation: CMPA, cow’s milk protein allergy.

The model simulates a cohort of 5-month-old community-based infants with IgE-mediated symptoms of CMPA, who are at risk of developing AM (eczema, asthma, rhinoconjunctivitis, or urticaria), but who can also become symptom-free.^17^ These health states were modeled as mutually exclusive and exhaustive, with annual probabilities of belonging to each health state adding up to 1. Management costs of CMPA, such as those for healthcare and dietetic replacements, were assigned to infants in each health state and were aggregated over successive years. Because we had no information of the number of children having multiple symptoms, we specifically modeled children’s main AM. We assumed that mortality due to CMPA or hypoallergenic formula intake would not differ among cohorts and have therefore excluded it from the analysis. A previously published simplified model structure is presented in **Figure 1**.^22^

### Model Inputs

**Allergic manifestations and tolerance to CMP:** The likelihood of AM and acquired tolerance to CMP were based on a prospective cohort study comparing AAF, EHCF+LGG, EHWF, RHF, and SF over a 36-month period.^17^ At the time of writing, this is the only trial providing a direct comparison between the relevant hypoallergenic formulas available in Indonesia and reporting on AM and probability of CMP tolerance over a 3-year follow-up. The systematic review by Strozyk et al,^24^ summarizing evidence on the efficacy of hydrolyzed formulas, confirms this, as it did not find additional studies expanding on the direct or indirect comparison of the most common hypoallergenic formulas. Nocerino et al^17^ recruited 365 non-breastfed infants (73 per comparator) below 1 year of age and suspected to have IgE-mediated CMPA. At enrollment in the trial, all children were established on hypoallergenic milk formula for 15 to 30 days and were symptom-free. All children were also referred to a tertiary specialist center and were complying with a cow’s milk–free diet. IgE-mediated CMPA status was confirmed at baseline and every 12 months during the planned clinical assessment and data collection sessions. Further data were collected on the frequency of AM, diet status, and compliance to the hypoallergenic formula prescribed. Children were subject to an elimination provocation test and a skin prick test to investigate cow’s milk tolerance status. Parents were instructed to contact the tertiary center in the event of allergic symptoms so that the cause of the reaction could be determined. The researchers collected data on the presence of atopic eczema, allergic rhinoconjunctivitis, allergic urticaria, and allergic asthma. The effect of the formula was assessed using binomial regression on the outcomes of interest. The authors considered the role of sex, duration of breastfeeding (≥2 months), weaning, number of siblings, family risk of allergy, passive smoking, maternal smoking status during pregnancy, and exposure to pets as confounders, having adjusted for these in the regression model. Statistical significance used an α less than 0.0125. A more detailed explanation of the study protocol and methodology are provided in the original publication.^17^ The efficacy parameters used in the model (annual probability of each CMPA symptom, of being symptom-free and of acquiring cow’s milk tolerance) were previously published.^22^

### Resource Use and Costs

To estimate resource use in the clinical management of CMPA, a survey based on clinician experience was designed in collaboration with Indonesian clinicians and applied to 5 general pediatricians, 5 pediatric gastroenterologists, and 5 pediatric allergists accustomed to treating children with CMPA. The interviewed clinicians were distributed in several cities around the archipelago. In Indonesia, general pediatricians are usually the first point of contact for children with CMPA symptoms and are responsible for initial management and referral to other subspecialties.^12,13^ The anonymized market survey elicited the typical treatment practices for children with CMPA to derive average annual number of medical services (eg, pediatric office visits, emergency department visits, hospital admissions, and referrals to other specialists), allergy tests, laboratory investigations, imaging tests, and the use of prescription drugs and dietetic replacements required to treat symptomatic children. Both general CMPA management and specific atopic manifestations were considered in the survey, according to the child’s age and time since treatment initiation. The unitary costs of appointments and examinations in the private practice were also elicited from the clinician survey. Interviewed experts signed a confidentiality agreement and received an honorarium for their participation.

Hypoallergenic formula requirements during the first 6 months of age were based on the EHCF+LGG formulary decision guide (876 mL/day, estimated from an average of 10 cans per month).^25^ Hypoallergenic milk formula posology for children with CMPA over the age of 6 months was collected in the clinician survey (784 mL/day from 6 to 12 months and 547 mL/day after 12 months).

At CMPA presentation (year 1 only), the resources used to diagnose and manage the initial symptoms of CMPA were imputed to all children. We assumed that the incidence of urticaria symptoms in years 2 and 3 would be due to accidental exposure to cow’s milk or AM to other foods (as part of the allergic march) and would be accompanied by gastrointestinal symptoms. Packed lunches and CMP-free supplements were assumed to be required 5 days per week. The estimated amount of health resources used for CMPA manifestations in the model are presented in the **Online Supplementary Material (Table S1)**.

Costs were obtained by multiplying the average number of resources per year by the unitary costs. The average number of resources per year was based on the simple averages from all respondents, as well as the unitary costs of appointments and exams in the private practice. The unitary cost of an accident and emergency attendance was based on a private hospital in Jakarta, including the admission, medical appointment, and 1 treatment administration. The cost of a hospital admission was assumed as the average price of first-class tariffs for hospitalization in Private Hospitals A due to compulsive nutrition disorders (INA-CBG F-4-18-i) as a proxy to the average cost at a private hospital.

Unit costs of milk formulas were based on the arithmetic mean price per 100 mL of reconstituted milk per milk formula category. Unit costs for hypoallergenic milk formulas and of dietetic replacements such as CMP-free snacks were based on retail outlets and on online pharmacies in Indonesia. Individual prices for hypoallergenic milk formulas are presented in the **Online Supplementary Material (Table S2)**.^26–31^ The cost of a packed lunch was estimated by the clinical experts. Unit costs of prescribed drugs were based on official listed prices.^32^ The cost of emollients without corticosteroids, eye drops, spacers for inhaled medicines, and adrenaline autoinjectors were based on online pharmacy prices in Indonesia.^33–36^ The cost of a trip to the clinical appointment was based on data from Botteman et al^37^ and adjusted to 2020 prices.^37,38^ The unit costs used to populate the model and its respective sources are shown in **Table 1**. Estimated costs were discounted at 3% rate after year 1, according to the Indonesian guidelines for the economic evaluation of healthcare technologies.^39^

**Table 1.** Unit Costs of Healthcare Resources and Nutrition

	**Unit Cost (IDR)**	**Source**
Appointments		
General pediatrician (follow-up visit)	196 875	Expert panel
Pediatric allergist	293 750	
Pediatric gastroenterologist	300 000	
Dermatologist	234 375	
Pediatric nutritionist	281 250	
Pediatric pulmonologist	281 250	
Accident and emergency attendance	1 570 000	Private hospital in Jakarta (admission, appointment, and treatment administration)
Hospital admission^a^	9 735 740	(INA-CBG F-4-18-I)^40^
Allergy and laboratory tests		
Skin prick	1 070 000	Expert panel
IgE-specific	1 619 467	
IgE total	548 000	
Elimination provocation test	290 000	
Fecalysis	182 250	
Complete blood count	150 000	
Fecal occult blood test	207 000	
Peripheral blood test	47 000	
Mantoux test	500 000	
Endoscopy	2 500 000	
Chest x-ray	228 636	
Sinus x-ray	353 000	
USG abdomen	350 000	
Spirometry	265 000	
Prescription drugs		
Emergency food allergy kit		
H_1_ antihistamines	50 000	MIMS,^32^ Bukalapak^33^
H_1_ antihistamines + adrenaline autoinjector	2 550 000	
Emergency asthma kit		
β_2_ + spacer	271 500	MIMS,^32^ Lazada^35^
β_2_ + spacer + oral corticosteroid	536 833	
Inhaled corticosteroid (cost per month)	61 788	MIMS^32^
Oral antihistamines (cost per month)	44 388	
Nasal corticosteroids	113 248	
Leukotriene antagonist	230 059	
Emollients (cost per mL)		
Atopiclair^®^	6959	Hdmall^34^
Ceradan^®^	6279	K24klik^36^
Sebamed^®^	1500	Lazada^35^
Topical corticosteroids (cost per tube)		
Elocon^®^ 0.1% (10 g)	66 200	MIMS^32^
Dermacoid^®^ 1% (10 g)	123 591	Hdmall^34^
Hydrocortisone 2.5% (Calacort^®^) (5 g)	17 000	MIMS^32^
Calcineurin inhibitor	801 490	Lazada^35^
Oral corticosteroids	93 037	Hdmall^34^
Eye drops (eg, sodium cromoglicate)	64 644	Hdmall,^34^ K24klik^36^
Diet		
CM-free packed lunch	15 000	Expert opinion
CM-free snack	10 500	Tokopedia^31^
Transportation (cost per trip)	47 288	Botteman et al,^37^ The World Bank Data^38^
Milk formulas^b^		
AAF (per 100 mL of reconstituted milk)	13 770	Raja Susu^29^
EHCF+LGG (per 100 mL of reconstituted milk)	13 019	MeadJohnson Indonesia^26^
EHWF (per 100 mL of reconstituted milk)	7434	Raja Susu^29^
SF (per 100 mL of reconstituted milk)	3251	Kalkare,^27^ KLIC Indomaret,^28^ Raja Susu,^29^ Shopee Indonesia,^30^ Tokopedia^31^

Abbreviations: AAF, amino acid–based formula; β_2_, beta-2 adrenergic receptor agonist; CM, cow’s milk; EHCF+LGG, extensively hydrolyzed casein formula containing *Lactobacillus rhamnosus* Gorbach Goldin; EHWF, extensively hydrolyzed whey formula; H1 receptor, histamine receptor; IDR, Indonesian rupiah; IgE,

immunoglobulin E; NHS, National Health Service; SF, soy formula; USG, ultrasonography.

^a^The cost of a hospital admission was calculated as the average price of first-class tariffs for hospitalization in Private Hospitals A (specialized) due to compulsive nutrition disorders, calculated as the average of costs per admission due to various nutritional disorders in children with severity level 1 in 2018, and admissions with a very short duration.

^b^Milk formula unit costs are presented in **Supplementary Table S2**.

### Measures of Effect

This analysis used the probability of acquiring cow’s milk tolerance and the absence of AM of CMPA as the main measures of hypoallergenic formula efficacy. The likelihood of being free from AM was estimated as the inverse of the probability of having any AM at the end of the 3-year study period.^17,19^ The likelihood of being cow’s milk–tolerant was directly sourced from Nocerino et al.^17^ Time free from symptoms and time being tolerant to CMP were also analyzed, considering the 3-year time horizon since treatment initiation. Estimating preference-based measures of quality of life in children in health economic evaluation is complex, particularly in children under the age of 5^41^; therefore, quality-adjusted life-years (QALYs) were considered inappropriate for this analysis. Health consequences were discounted at a 3% rate in years 2 and 3 of the analysis.^39^

### Sensitivity Analyses

As both effect and costs parameters are subject to uncertainty, the robustness of the results was assessed by one-way and probabilistic sensitivity analysis. Each base case input was varied using 95% confidence intervals to assess which had the most impact on results. These one-way sensitivity analyses are reported in a tornado diagram (**Supplemental Figure S1**). Two additional scenarios implementing a 30% increase or decrease in healthcare resources utilization were run to explore heterogeneity arising from experts’ opinions. Probabilistic sensitivity analysis was conducted by applying common probability distributions to all model inputs, which were then resampled using 1000 Monte Carlo simulations.^42^ The likelihoods of AM and being symptom-free were sampled from Dirichlet distributions, and a beta distribution was applied to the probability of being tolerant to CMP, based on data reported by Nocerino et al.^17^ An uniform distribution was applied to costs with a standard error of 10%, due to the lack of data for its variance.

## RESULTS

### Base Case

Infants fed with EHCF+LGG had a higher probability of being symptom-free and tolerant to CMP after 3 years in the base case. At this time horizon, EHCF+LGG was also associated with increased time free from allergic symptoms and time tolerant to cow’s milk (**Table 2**).

**Table 2.** Results for Measures of Effect, at 3 Years (Discounted)

	**Formula**			
**Effect at 3 Years**	**SF**	**EHCF+LGG**	**EHWF**	**AAF**
Probability of being symptom-free^a,b^	0.527	0.710	0.539	0.646
Life-years without symptoms	1.712	2.441	1.857	1.624
Probability of cow’s milk tolerance^b,c^	0.376	0.762	0.401	0.181
Life-years being tolerant to CMP	0.738	1.795	0.901	0.293

Abbreviations: AAF, amino acid-based formula; CMP, cow’s milk protein; EHCF+LGG, extensively hydrolyzed casein formula containing *Lactobacillus rhamnosus* Gorbach Goldin; EHWF, extensively hydrolyzed whey formula; SF, soy formula.

^a^Calculated as 1 minus the sum of the annual probabilities of urticaria, eczema, asthma, and rhinoconjunctivitis for that cycle.

^b^Probabilities previously published in Martins R, Connolly M, Minshall E. *J Health Econ Outcomes Res*. 2021;8(2):14-25.^22^

^c^Cumulative probabilities.

Costs per healthcare category per average child receiving each milk formula are presented in **Table 3**. For all comparators, the expenditure on infant formula was responsible for the highest cost in managing CMPA. On average, it accounted for 67% of total costs (with a minimum of 47% for SF and a maximum of 79% for AAF). Dietetic replacement options were the second highest cost component, representing 14% to 36% of total costs in EHCF+LGG and SF, respectively. Children receiving SF were predicted to incur lower total costs due to the lowest cost of milk formula. However, when formula costs were excluded, EHCF+LGG was predicted to have the lowest total costs, a reduction of 38%, 42%, and 49% when compared with EHWF, SF, and AAF, respectively. Diagnostic tests, specialists’ visits, hospital admissions, accident and emergency attendances, and prescription drugs added to approximately 10% of total costs.

**Table 3.** Total Costs per Healthcare Resource (Discounted)

	**Costs per Formula (IDR)**			
**Resource**	**SF**	**EHCF+LGG**	**EHWF**	**AAF**
Infant formula	14 470 948	32 460 186	30 679 782	72 540 868
Diet	11 300 979	6 054 528	10 488 198	13 507 582
Diagnostics	1 703 539	1 423 272	1 646 336	1 757 300
Specialist visits	907 735	623 878	867 951	948 082
Hospital admissions	623 852	521 039	681 391	608 177
Pediatrician visits	589 946	451 171	586 934	601 397
Transportation	315 223	225 574	306 079	328 662
Prescribed drugs	867 507	168 546	759 865	988 132
A&E attendances	235 883	148 960	273 546	215 754
Nutritionist visits	104 103	104 103	104 103	104 103
**Total**	31 119 716	42 181 257	46 394 186	91 600 058

Abbreviations: AAF, amino acid–based formula; A&E, accident and emergency; CM, cow’s milk; EHCF+LGG, extensively hydrolyzed casein formula containing *Lactobacillus rhamnosus* Gorbach Goldin; EHWF, extensively hydrolyzed whey formula; IDR, Indonesian rupiah; SF, soy formula.

**Figure 2.**
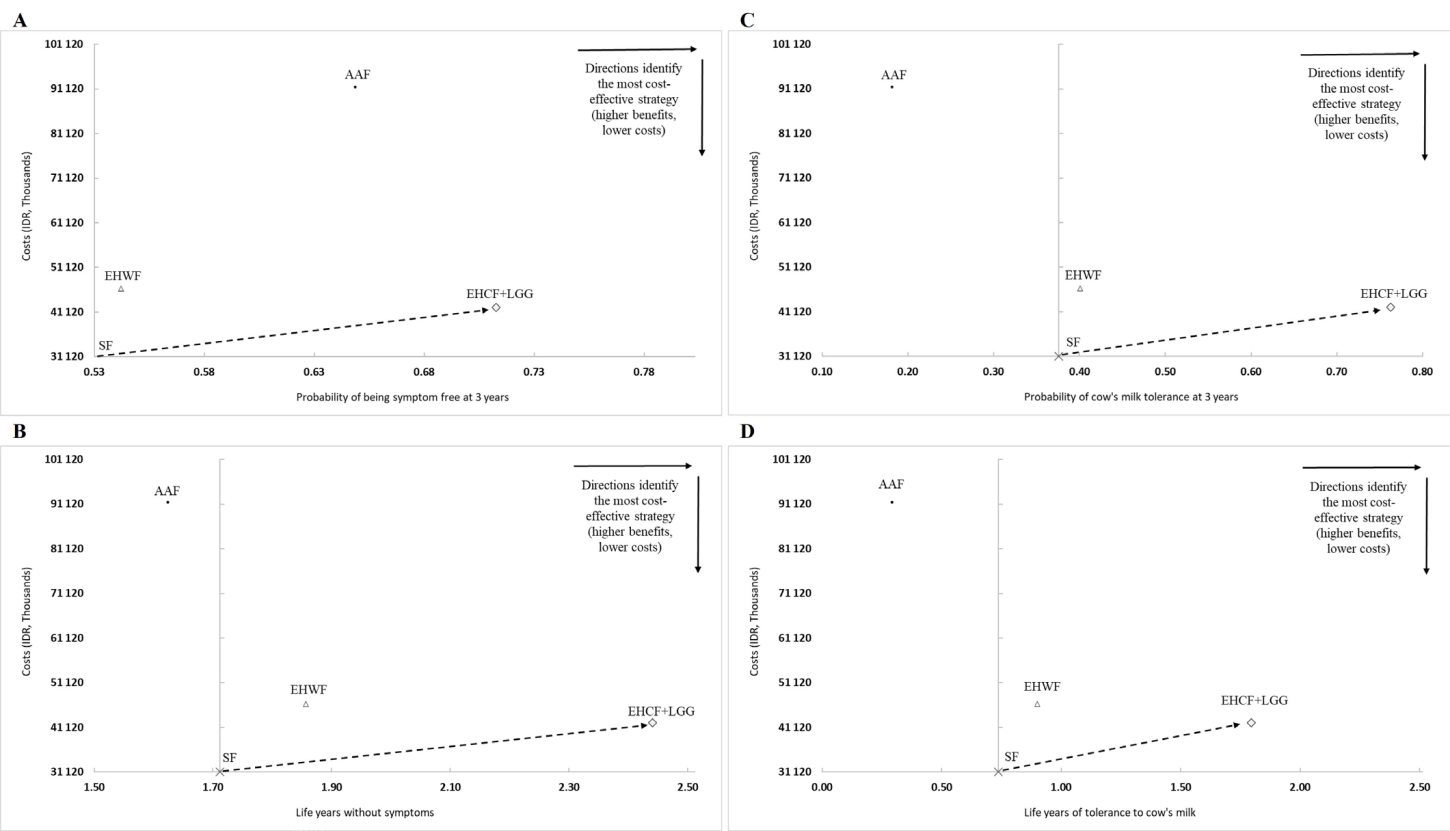
Base Case Deterministic Results Displayed on the Cost-Effectiveness Plane **(A)** Results for cost per probability of being symptom-free at 3 years. **(B)** Results for cost per life-year without symptoms at 3 years. **(C)** Results for cost per probability of being tolerant to cow’s milk at 3 years. **(D)** Results for cost per life-year with tolerance to cow’s milk at 3 years. Abbreviations: AAF, amino acid–based formula; EHCF+LGG, extensively hydrolyzed casein formula containing *Lactobacillus rhamnosus* Gorbach Goldin; EHWF, extensively hydrolyzed whey formula; IDR, Indonesian rupiah; SF, soy formula.

The average cost per measure of effect is presented for all formulas (**Table 4**). The model predicted that EHCF+LGG is the most cost-effective strategy for most measures of effect, with a lower ratio of cost per measure of effect. The exception is the cost per symptom-free child at year 3, for which SF is marginally lower than EHCF+LGG. **Figure 2** graphically represents overall costs and effects (for probabilities of being symptom-free and of being immunotolerant to cow’s milk, at 3 years) in a cost-effectiveness plane. Soy formula is represented at the origin as the least expensive milk formula available. Any formula represented to the right of the origin provides increased benefits when compared with SF; any formula represented upward is associated with an additional cost. The slope between 2 milk formulas represents the incremental cost-effectiveness ratio between them (the incremental costs required to gain an additional measure of effect). The dashed arrows in **Figure 2** show the additional cost per average child receiving EHCF+LGG per unit of effect, when compared with SF (Indonesian rupiahs [IDR] 60 471 964 per symptom-free child, IDR 15 172 555 per life-year without symptoms, IDR 28 632 469 per additional immunotolerant child, and IDR 10 467 410 per life year being tolerant to CMP). The strategy of using EHCF+LGG was considered dominant for all assessed outcomes when compared with EHWF and AAF, being associated with lower costs and added benefits. A full incremental analysis of all comparators is presented in the **Online Supplementary Material (Table S3)**.

**Table 4.** Base Case Deterministic Results (Discounted)

	**Cost per Unit of Effect (IDR)**			
**Measure of Effect at 3 Years**	**SF**	**EHCF+LGG**	**EHWF**	**AAF**
Symptom-free child	59 069 109	59 430 656	85 057 265	141 893 355
Life-years without symptoms	18 176 783	17 279 555	24 983 401	56 416 627
Cow’s milk–immunotolerant child	82 735 832	55 322 434	115 814 075	504 248 582
Life-years being tolerant to CMP	42 156 854	23 499 976	51 518 220	312 404 048

Abbreviations: AAF, amino acid–based formula; CMP, cow’s milk protein; EHCF+LGG, extensively hydrolyzed casein formula containing *Lactobacillus rhamnosus* Gorbach Goldin; EHWF, extensively hydrolyzed whey formula; IDR, Indonesian rupiah; SF, soy formula.

### Sensitivity Analyses

**One-way sensitivity analyses**: Varying the 10 most influential parameters according to their 95% confidence intervals showed that the model results showed a higher variation when the annual probabilities of acquiring tolerance to CMP and the probability of being symptom-free at 3 years were changed. However, varying all these parameters in the one-way sensitivity analysis did not change the model conclusions. Due to the up-front price of milk formulas only, SF remains the lower-cost option; nonetheless, receiving EHCF+LGG was associated with lower costs and added benefits compared with EHWF and AAF. Tornado diagrams comparing the incremental cost-effectiveness ratios of EHCF+LGG vs EHWF are depicted in **Supplementary Figure S1**.

The scenarios increasing or decreasing healthcare resource utilization by 30% did not change the conclusion of the analysis, as this variation did not contribute to the incremental difference in costs between strategies.

**Probabilistic sensitivity analysis:** The results of the probabilistic sampling are plotted in the cost-effectiveness plane depicted in **Supplementary Figure S2**. From the plotted simulations, we can conclude that when uncertainty is considered, the increased benefit for those receiving EHCF+LGG is clear for most health outcomes. Results do not seem so clear for the probability of being symptom-free at 3 years, in which case AAF showed a better outcome in 17% of the simulations, always at a higher cost. Soy formula provided the lowest costs, regardless of the outcomes.

## DISCUSSION

The benefits of breast milk have been widely recognized by the scientific community as the best for infants.^13,43^ Nonetheless, there are situations in which breast milk is insufficient or unavailable, or parents choose not to breastfeed. When infants are allergic to cow’s milk, recommendations are that breast milk may be replaced or supplemented with hypoallergenic milk formulas.^12,13,44,45^ Furthermore, as the choice of hypoallergic milk formula may influence AM or related conditions, the choice of formula is important for improving outcomes and reducing future healthcare visits.

In 2018, domestic private health expenditures from households, corporations, and nonprofit organizations corresponded to 50.3% of all Indonesian healthcare spending, with 34.9% of all healthcare expenditures coming directly from out-of-pocket payments by households.^46,47^ Nonprescription healthcare products are commonly not reimbursed, representing a substantial financial burden for patients and families. It is therefore crucial that families’ choice of hypoallergenic milk formula uses strict cost-effectiveness criteria to maximize health improvements in face of available resources. With this in mind, we have conducted this analysis from the private market (families’) perspective using standard cost-effectiveness methods adopted for use in Indonesian policy analysis since 2003.^39^

We could find only 1 economic evaluation comparing the use of a partially hydrolyzed formula in infants at high-risk of atopic dermatitis conducted in the Indonesian setting, based on the GINI study.^37,48^ However, to our knowledge, the present study is the first to directly compare the cost-effectiveness of the main hypoallergenic milk formulas available in Indonesia in treating children with symptomatic CMPA for up to 3 years. Based on the results of a recent clinical trial reporting on AM and CMPA tolerance,^17^ our modeled evaluation predicted that children receiving EHCF+LGG were associated with a faster improvement of CMPA symptoms and acquisition of cow’s milk tolerance, leading to a more rapid reduction in healthcare needs and formula utilization compared with alternative hypoallergenic milk formulas. Due to its low acquisition cost, SF remained at the cost-effectiveness frontier, but this should be interpreted with caution. First, SF is not usually recommended as first-line management of CMPA.^12,13^ This is due to its allergenicity, being poorly tolerated by 8% to 10% of infants with CMPA.^49,50^ Second, low formula costs deflate the total costs associated with SF, masking the increase in healthcare resource consumption due to increased symptoms in children.

The current study carries limitations that should be discussed. First, the effectiveness data rely on a nonrandomized prospective study from a single European country. Nevertheless, the study was sufficiently powered to detect differences in the incidence of AM between comparator arms, which was one of the main outcomes of the current model. Results were adjusted for confounding using a binary regression model. Further, because the probability of tolerance to CMP was a secondary outcome, the study may not have been powered to detect a difference between comparators.^17^ However, study results are in line with previous evidence of the effect of hypoallergenic formulas on the incidence of AM and acquisition of immune tolerance and were therefore deemed appropriate to inform treatment efficacy.^15,16,24,48,51–56^ Additionally, to the best of our knowledge and a published literature review in this topic area, there is no randomized study comparing the formula products relevant for the analysis in Indonesia.^24^ Resource estimation was obtained from 5 general pediatricians, 5 pediatric allergists, and 5 pediatric gastroenterologists practicing in Indonesia, which comprises a limited sample. Nonetheless, we have challenged the face validity of the resulting inputs by subjecting them to scrutiny by experienced Indonesian clinicians. In addition, we have conducted sensitivity analyses, which demonstrated robustness in our results.

Our analysis does not use nonclinical outcomes such as QALYs or disability-adjusted life-years, commonly included in cost-effectiveness analyses.^39^ Instead, we use the likelihood of being symptom-free or tolerant to CMP and the number of years lived without symptoms or with acquired cow’s milk immunotolerance. Reasons not to include QALYs or disability-adjusted life-years in the current analysis include the methodological challenges of measuring utilities in children younger than 5 years old and the large variation in the incidence, intensity, and duration of AM among children.^41^ Also, symptoms of CMPA and associated AM impact both children’s and families’ well-being. Therefore, clinical outcomes such as acquired tolerance and the absence of AM were deemed meaningful to clinicians and families.

As with the study sourced for the efficacy data, the current model did not account for any adverse events related to the milk formulas.^17^ Immune tolerance is likely to improve child development, enhance families’ well-being, and decrease costs on healthcare and infant formulas. The impact on child’s development and on families’ wellbeing was not directly considered in the current model. It is therefore expected that the real burden of CMPA and the benefits from hypoallergenic formulas had been underestimated in this study.

## CONCLUSION

Compared with the common hypoallergenic formulas, EHCF+LGG is predicted to be the most cost-effective strategy when compared with both EHWF and AFF. Although SF is a lower-cost option, EHCF+LGG proved to be more effective and provide savings in healthcare resources in terms of clinical appointments, medical exams, and prescribed drugs as well as dietetic replacements. Acknowledging the breakdown of costs alongside incremental benefits should allow clinicians to realize the impact of their recommendations and empower families to make more informed decisions about their out-of-pocket expenses related to CMPA. Although our results are subject to some data limitations, we believe they can inform the choice of hypoallergenic formula for non-breastfed children in Indonesia.

### Author Contributions

A.T.P. and R.M. participated in the model design and in the expert survey development, wrote the original draft manuscript, and reviewed and edited the manuscript. M.P.C. participated in the conceptualization of the model, reviewed the expert survey and manuscript, and was responsible for project administration. B.H. and Z.M. reviewed the expert survey and the manuscript. S. reviewed the expert survey, provided local market data, and reviewed the manuscript.

### Disclosures

A.T.P., R.M., and M.P.C. are researchers for Global Market Access Solutions and were funded by Mead Johnson Nutrition Indonesia. B.H. and Z.M. were paid advisors to Mead Johnson Nutrition, Indonesia. S. is an employee of Mead Johnson Indonesia. A.T.P., R.M., M.P.C., B.H., and Z.M. specifically have no financial interests in the commercial operations of Mead Johnson Nutrition, Indonesia and related organizations. The content of this analysis represents the authors’ views and may not reflect those of the sponsoring organization. The authors have no other affiliations or conflicts of interest to report.

## Supplementary Material

Online Supplementary Material
