# Supplementary material for: Managing Cow’s Milk Protein Allergy in Indonesia: A Cost-effectiveness Analysis of Hypoallergenic Milk Formulas from the Private Payers’ Perspective
Source: J Health Econ Outcomes Res. 2022 Sep 7;9(2):77–85. doi: 10.36469/001c.36407 (PMC9452772; doi:10.36469/001c.36407)
Supplement: Online Supplementary Material [file jheor_2022_9_2_36407_99201.pdf]

Journal of Health Economics and Outcomes Research

Online Supplementary Material

Managing Cow's Milk Protein Allergy in Indonesia: A Cost-effectiveness Analysis of Hypoallergenic Milk Formulas From the Private Payers' Perspective. *JHEOR*. 2022;9(2):77-85. doi:10.36469/jheor.2022.36407

Table S1: Estimates of Resource Use in the Model (Survey of Clinical Experts)

Table S2: Milk Formula Unit Costs

Table S3: Base Case Deterministic Results: Incremental Cost-effectiveness Ratios

Figure S1: Tornado Diagrams for EHCF + LGG vs EHWF

Figure S2: Probabilistic Results Displayed on Cost-effectiveness Planes

This supplementary material has been provided by the authors to give readers additional information about their work.

This is an open-access article distributed under the terms of the Creative Commons Attribution 4.0 International License (CCBY-4.0). View this license's legal deed at http://creativecommons.org/licenses/by/4.0 and legal code at http://creativecommons.org/licenses/by/4.0/legalcode for more information.

Table S1. Estimates of Resource Use in the Model (Survey of Clinical Experts)

	Mean Units per Average Child, by Symptom and Time Since Treatment Initiation												
	**CMPA/Urticaria**	**Eczema**			**Acute Urticaria and Other Signs**			**Asthma-like Symptoms**			**Rhinoconjunctivitis**		
	**Year 1**	**Year 1**	**Year 2**	**Year 3**	**Year 1**	**Year 2**	**Year 3**	**Year 1**	**Year 2**	**Year 3**	**Year 1**	**Year 2**	**Year 3**
General pediatrician visits	2.17	2.37	0.27	0.02	1.08	0.25	0.17	2.15	1.31	0.96	0.00	0.00	0.00
Pediatric allergist visits	0.75	0.44	0.12	0.05	1.87	0.27	0.05	0.73	0.06	0.23	0.00	0.00	0.11
Pediatric gastroenterologist visits	1.17	0.44	0.12	0.05	0.26	0.38	0.14	0.17	0.31	0.27	0.00	0.00	0.00
Dermatologist visits	—	0.44	0.12	0.05	—	—	—	—	—	—	—	—	—
Pediatric pulmonologist visits	—	—	—	—	—	—	—	0.84	0.28	0.16	—	—	—
Nutritionist visits	0.38	—	—	—	—	—	—	—	—	—	—	—	—
Accident and emergency attendances	0.07	0.00	0.00	0.00	0.05	0.02	0.02	0.69	0.41	0.20	0.00	0.00	0.00
Hospital admissions	0.05	—	—	—	0.03	0.00	0.00	0.15	0.05	0.03	—	—	—
Emergency food allergy kit prescription	—	—	—	—	—	—	—	—	—	—	—	—	—
H_1 antihistamines	54%	—	—	—	86%	86%	86%	—	—	—	—	—	—
H_1 antihistamines and adrenaline autoinjector	1%	—	—	—	2%	2%	2%	—	—	—	—	—	—
Emergency asthma kit prescription	—	—	—	—	—	—	—	—	—	—	—	—	—
β_2 and spacer	—	—	—	—	—	—	—	44%	44%	44%	—	—	—
β_2, spacer, and oral corticosteroid	—	—	—	—	—	—	—	24%	24%	24%	—	—	—
Emollients prescription	—	—	—	—	—	—	—	—	—	—	—	—	—
Atopiclair® (mL/day)	—	7.50	6.50	4.60	—	—	—	—	—	—	—	—	—
Ceradan® (mL/day)	—	7.20	3.60	2.00	—	—	—	—	—	—	—	—	—
Sebamed® (mL/day)	—	3.00	1.50	1.50	—	—	—	—	—	—	—	—	—
Duration of treatment (mo)	—	2.56	0.91	0.54	—	—	—	—	—	—	—	—	—
Topical corticosteroid prescription (tubes/mo)	—	1.71	0.56	0.47	—	—	—	—	—	—	—	—	—
Elocon® 0.1%	—	1.14	1.00	0.75	—	—	—	—	—	—	—	—	—
Dermacoid® 1%	—	2.00	0.33	0.33	—	—	—	—	—	—	—	—	—
Hydrocortisone 2.5% (Calacort®)	—	2.00	0.33	0.33	—	—	—	—	—	—	—	—	—
Duration of treatment (mo)	—	0.19	0.04	0.01	—	—	—	—	—	—	—	—	—
Oral antihistamine prescription (average time on treatment, mo)	—	0.22	0.03	0.02	0.14	0.03	0.02	—	—	—	0.45	0.23	0.23
Calcineurin inhibitor (average time on treatment, mo)	—	0.04	0.01	0.01	—	—	—	—	—	—	—	—	—
Oral corticosteroid prescription (average time on treatment, mo)	—	—	—	—	0.04	0.02	0.01	—	—	—	—	—	—

Table S1. Estimates of Resource Use in the Model (Survey of Clinical Experts), *cont'd*

	**Mean Units per Average Child, by Symptom and Time Since Treatment Initiation**												
	**CMPA/Urticaria**	**Eczema**			**Acute Urticaria and Other Signs**			**Asthma-like Symptoms**			**Rhinoconjunctivitis**		
	**Year 1**	**Year 1**	**Year 2**	**Year 3**	**Year 1**	**Year 2**	**Year 3**	**Year 1**	**Year 2**	**Year 3**	**Year 1**	**Year 2**	**Year 3**
Inhaled corticosteroids prescription (average time on treatment, mo)	—	—	—	—	—	—	—	0.29	0.02	0.01	—	—	—
Nasal corticosteroids prescription (average time on treatment, mo)	—	—	—	—	—	—	—	—	—	—	0.12	0.03	0.06
Leukotriene antagonist prescription (average time on treatment, mo)	—	—	—	—	—	—	—	—	—	—	0.00	0.00	0.01
Eye drops prescription (average time on treatment, mo)	—	—	—	—	—	—	—	—	—	—	0.00	0.00	0.01
Percentage having packed lunch	17%	—	—	—	—	—	—	—	—	—	—	—	—
CMP-free snack (mean units/day)	1.69	—	—	—	—	—	—	—	—	—	—	—	—
Skin prick test	0.27	0.07	0.07	0.07	0.38	0.38	0.38	—	—	—	—	—	—
IgE-specific tests	0.24	0.05	0.05	0.05	0.49	0.49	0.49	0.13	0.13	0.13	—	—	—
IgE total	—	—	—	—	—	—	—	—	—	—	0.04	0.04	0.04
Elimination provocation test	0.76	—	—	—	0.73	0.73	0.73	—	—	—	—	—	—
Chest x-ray	0.02	—	—	—	—	—	—	0.48	0.48	0.48	—	—	—
Sinus x-ray	—	—	—	—	—	—	—	—	—	—	0.11	0.11	0.11
USG abdomen	0.04	—	—	—	0.03	0.03	0.03	—	—	—	—	—	—
Fecalysis	0.78	—	—	—	—	—	—	—	—	—	—	—	—
Fecal occult blood test	0.71	—	—	—	—	—	—	—	—	—	—	—	—
Endoscopy	0.01	—	—	—	—	—	—	—	—	—	—	—	—
Peripheral blood test	0.13	—	—	—	—	—	—	—	—	—	—	—	—
Spirometry	—	—	—	—	—	—	—	0.10	0.10	0.10	—	—	—
Complete blood cell count	—	—	—	—	—	—	—	0.14	0.14	0.14	0.04	0.04	0.04
Mantoux test	—	—	—	—	—	—	—	0.07	0.07	0.07	—	—	—

Abbreviations: β_2, beta-2 adrenergic receptor agonist; CMP, cow's milk protein; CMPA, cow's milk protein allergy; H1, histamine receptor; IgE, immunoglobulin E; USG, ultrasonography.

Table S2. Milk Formula Unit Costs

Formula Type	Brand	Weight of Powder per 100 mL Milk (g)	Price per 400 g can (IDR)	Price per 100 mL Reconstituted Milk (IDR)	Average Price per 100 mL Reconstituted Milk (IDR)	Source
EHCF + LGG	1	13.6	382 900	13 019	13 019	MeadJohnson Nutrition[1]
EHWF	1	12.6	236 000	7434	7434	Raja Susu[2]
AAF	1	13.5	449 246	15 162	13 770	Raja Susu[2]
	2	13.5	340 000	11 475		
	3	21.9	268 000	14 673		
SF	1	13.5	39 222	1324	3251	Raja Susu,[2] Shopee Indonesia,[3] KLIC Indomaret,[4] Tokopedia,[5] Kalkare[6]
	2	13.8	123 200	4250		
	3	13.8	120 667	4163		
	4	13.2	64 000	2112		
	5	13.2	38 509	1271		
	6	12.9	41 886	1351		
	7	13.2	126 200	4165		
	8	14.5	119 333	4326		
	9	14.5	86 750	3145		
	10	14.4	34 229	1232		
	11	17.7	82 286	3641		
	12	17.7	76 800	3398		
	13	14.4	123 800	4466		
	14	14.4	134 648	4857		
	15	16.0	114 667	4587		
	16	16.0	109 333	4373		
	17	20.0	59 429	2971		
	18	23.1	50 000	2888		

Abbreviations: AAF, amino acid–based formula; EHCF + LGG, extensively hydrolyzed casein formula containing *Lactobacillus rhamnosus* Gorbach Goldin (EHCF+ LGG); EHWF, extensively hydrolyzed whey formula; IDR, Indonesian rupiah; SF, soy formula.

Table S3. Base Case Deterministic Results: Incremental Cost-effectiveness Ratios

	**Incremental Results**		
	**EHCF+LGG (vs SF)**[a]	**EHWF (vs EHCF+LGG)**[b]	**AAF (vs EHCF+LGG)**[b]
Incremental costs (IDR)	11 061 541	4 212 929	49 418 801
Incremental effects at 3 years			
Probability of being symptom-free	0.183	-0.171	-0.064
Life-years without symptoms	0.729	-0.584	-0.817
Probability of being cow's milk immunotolerant	0.386	-0.362	-0.581
Life-years with cow's milk tolerance	1.057	-0.894	-1.502
Measure of effect, at 3 years	**ICER (IDR)**[c]		
Incremental cost per symptom-free child	60 471 964	Dominated[d] by EHFC+LGG	Dominated[d] by EHFC+LGG
Incremental cost per life-year without symptoms	15 172 555		
Incremental cost per cow's milk immunotolerant child	28 632 469		
Incremental cost per life-year with cow's milk tolerance	10 467 410		

Abbreviations: AAF, amino acid–based formula; EHCF+LGG, extensively hydrolyzed casein formula containing *Lactobacillus rhamnosus* Gorbach Goldin (EHCF + LGG); EHWF, extensively hydrolyzed whey formula; ICER, incremental cost-effectiveness ratio; IDR, Indonesian rupiah; SF, soy formula.
[a]EHCF+LGG is compared with the milk formula with lower costs (SF).
[b]EHWF and AAF are compared with EHCF+LGG, as this is the milk formula with second-lowest cost.
[c]Calculated as the difference in costs divided by the difference in effects.
[d]Dominated strategies have higher costs and lower effect.

Figure S1. Tornado Diagrams for EHCF+LGG vs EHWF[a]

A.

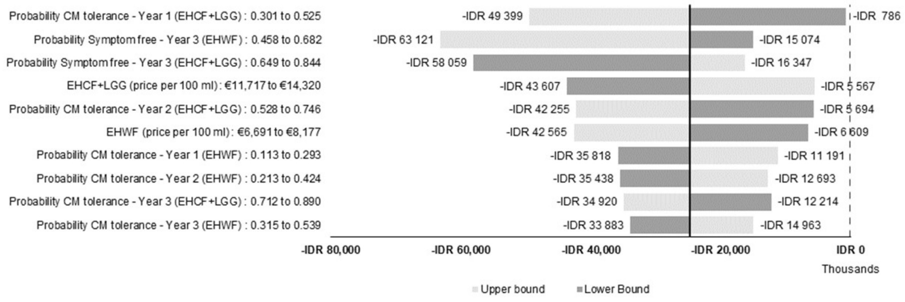

B.

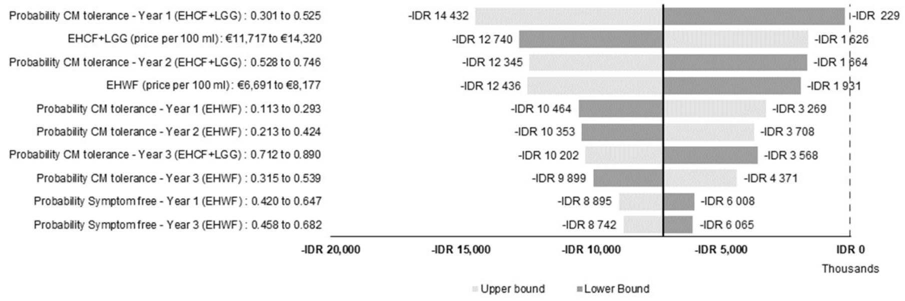

C.

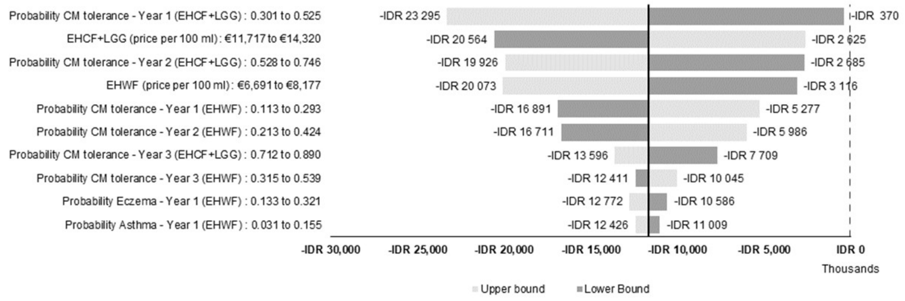

D.

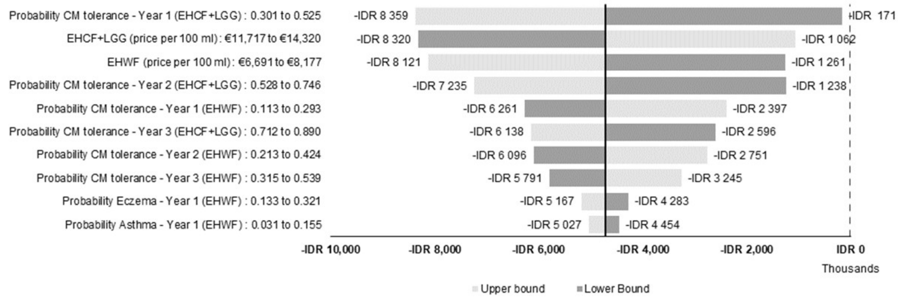

(**A**) Tornado diagram for cost per probability of being symptom-free at 3 years; (**B**) tornado diagram for cost per life-year without symptoms at 3 years; (**C**) tornado diagram for cost per probability of being tolerant to cow's milk at 3 years; (**D**) tornado diagram for cost per life-year with tolerance to cow's milk at 3 years.

Abbreviations: CM, cow's milk; EHCF+LGG, extensively hydrolyzed casein formula containing *Lactobacillus rhamnosus* Gorbach Goldin; EHWF, extensively hydrolyzed whey formula; IDR, Indonesian rupiah.

[a]EHWF was chosen for one-way sensitivity analysis presentation as it has shown to be the second-best option on both outcomes considered, after EHCF + LGG. Each tornado diagram depicts the variation around the incremental cost-effectiveness ratio (ICER): the incremental cost of patients receiving EHWF instead of EHCF+LGG divided by the incremental benefits.

Figure S2. Probabilistic Results Displayed on Cost-effectiveness Planes

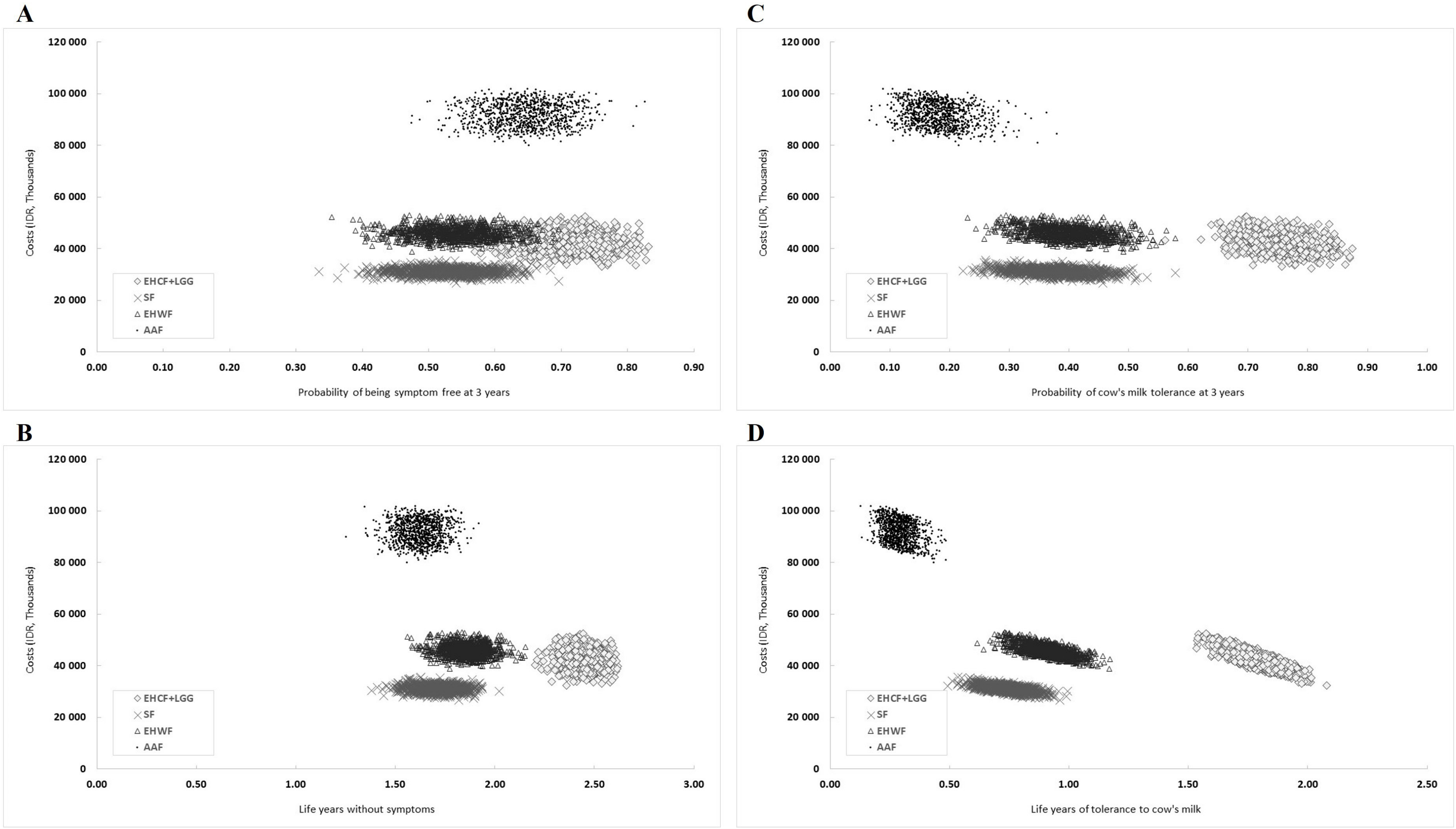

(**A**) Cost-effectiveness plane for the probability of being symptom-free at 3 years.
(**B**) Cost-effectiveness plane per life-year without symptoms at 3 years.
(**C**) Cost-effectiveness plane for the probability of being tolerant to cow's milk at 3 years.
(**D**) Cost-effectiveness plane per life-year with tolerance to cow's milk at 3 years.
Abbreviations: AAF, amino acid–based formula; EHCF+LGG, extensively hydrolyzed casein formula containing *Lactobacillus rhamnosus* Gorbach Goldin; EHWF, extensively hydrolyzed whey formula; IDR, Indonesian rupiah; SF, soy formula.
